# Blood coagulation abnormalities in multibacillary leprosy patients

**DOI:** 10.1371/journal.pntd.0006214

**Published:** 2018-03-22

**Authors:** Débora Santos da Silva, Lisandra Antonia Castro Teixeira, Daniela Gois Beghini, André Teixeira da Silva Ferreira, Márcia de Berredo Moreira Pinho, Patricia Sammarco Rosa, Marli Rambaldi Ribeiro, Monica Di Calafiori Freire, Mariana Andrea Hacker, José Augusto da Costa Nery, Maria Cristina Vidal Pessolani, Ana Maria Freire Tovar, Euzenir Nunes Sarno, Jonas Perales, Fernando Augusto Bozza, Danuza Esquenazi, Robson Queiroz Monteiro, Flavio Alves Lara

**Affiliations:** 1 Lab. of Cellular Microbiology, Oswaldo Cruz Institute, Oswaldo Cruz Foundation, Rio de Janeiro, Brazil; 2 Lab. of Conjunctive Tissue, Institute of Medical Biochemistry, Federal University of Rio de Janeiro, Rio de Janeiro, Brazil; 3 Lab. of Toxinology, Oswaldo Cruz Institute, Oswaldo Cruz Foundation, Rio de Janeiro, Brazil; 4 Department of Biology, Lauro de Souza Lima Institute, Bauru, Brazil; 5 Lab. Dr. Sérgio Franco, Hematology Department, DASA, Rio de Janeiro, Brazil; 6 Lab. of Leprosy, Oswaldo Cruz Institute, Oswaldo Cruz Foundation, Rio de Janeiro, Brazil; 7 Department of Critical Care, National Institute of Infectious Disease, Oswaldo Cruz Foundation, Rio de Janeiro, Brazil; 8 Lab. of Hemostasis and Poisons, Institute of Medical Biochemistry, Federal University of Rio de Janeiro, Rio de Janeiro, Brazil; University of Tennessee, UNITED STATES

## Abstract

**Background:**

Leprosy is a chronic dermato-neurological disease caused by *Mycobacterium leprae* infection. In 2016, more than 200,000 new cases of leprosy were detected around the world, representing the most frequent cause of infectious irreversible deformities and disabilities.

**Principal findings:**

In the present work, we demonstrate a consistent procoagulant profile on 40 reactional and non-reactional multibacillary leprosy patients. A retrospective analysis in search of signs of coagulation abnormalities among 638 leprosy patients identified 35 leprosy patients (5.48%) which displayed a characteristic lipid-like clot formed between blood clot and serum during serum harvesting, herein named ‘leprosum clot’. Most of these patients (n = 16, 45.7%) belonged to the lepromatous leprosy pole of the disease. In addition, formation of the leprosum clot was directly correlated with increased plasma levels of soluble tissue factor and von Willebrand factor. High performance thin layer chromatography demonstrated a high content of neutral lipids in the leprosum clot, and proteomic analysis demonstrated that the leprosum clot presented in these patients is highly enriched in fibrin. Remarkably, differential 2D-proteomics analysis between leprosum clots and control clots identified two proteins present only in leprosy patients clots: complement component 3 and 4 and inter-alpha-trypsin inhibitor family heavy chain-related protein (IHRP). In agreement with those observations we demonstrated that *M*. *leprae* induces hepatocytes release of IHRP *in vitro*.

**Conclusions:**

We demonstrated that leprosy MB patients develop a procoagulant status due to high levels of plasmatic fibrinogen, anti-cardiolipin antibodies, von Willebrand factor and soluble tissue factor. We propose that some of these components, fibrinogen for example, presents potential as predictive biomarkers of leprosy reactions, generating tools for earlier diagnosis and treatment of these events.

## Author summary

Hemostatic illnesses are frequently associated with acute and chronic infections. In the present work we demonstrated that leprosy patients developed hemostatic abnormalities, like the formation of an atypical lipid clot mass during sera harvesting, a phenomenon previously observed and never unraveled. We characterize the nature of the “leprosum clot”, formed during a protrombotic state developed by some patients. During the proteomic analysis of the leprosum clot we discovered a set of potential serum biomarkers to leprosy reactional episodes diagnosis, which at this moment is based only in clinical features. Taking together, our data suggest that leprosy patients are suffering from a procoagulant status, being beneficiated by the introduction of routine coagulation tests during their treatment, which will aloud physicians to prevent some of the acute clinical symptoms related with superficial vein thrombosis such as cyanosis and tissue necrosis observed during severe cases of leprosy reactional episodes.

## Introduction

Leprosy remains an important public health problem worldwide. Damage to peripheral nerves and bone absorption [1] results in the deformities and disability that are hallmarks of the disease. There are two separate aspects to the disease. The first aspect involves direct infection of Schwann cells [2] and macrophages [3] by *M*. *leprae* and systemic immune response against the infection [4]. The second aspect includes reactional episodes that may affect nearly 50% of patients [5]. In contrast with mild and slow direct effects due to the presence of bacilli in the cells, reactional episodes are acute and highly deleterious to tissues and the peripheral nervous system, frequently causing medical emergencies in this chronic disease. Clinically, type 2 reactional episodes are considered a necrotizing panvasculitis, presenting different ranges of endothelium inflammation, followed by cyanosis with necrotic-hemorrhagic lesions on the extremities and trunk in more severe cases, such as Lucio’s phenomenon [6, 7].

Hemostatic disorders are frequently associated with acute and chronic infections due to the fact that platelet functions, blood coagulation and fibrinolysis are intimately correlated with the immune system [8–10]. Platelet disorders have previously been described in lepromatous and tuberculoid leprosy patients as impairments in adhesiveness and aggregation to collagen [11]. It was already reported that patients developing erythema nodosum leprosum (ENL) present prolonged activated partial thromboplastin time (aPTT) with high fibrinogen and platelet titers [7], together with platelet activation [12]. Deep thrombophlebitis followed by multiple pulmonary embolism was already described in leprosy patients suffering ENL [13], as well as edema, a frequent complication of leprosy reactional episodes [14]. Decades before the development of an efficient treatment for leprosy, Rogers and colleagues observed that from a total of 101 leprosy patients, 9 of them died from coronary thrombosis, and 5 of them from cerebral vascular accident, indicating that advanced non-treated leprosy could be related to higher incidence of stroke [15].

With the success of WHO multidrug therapy implementation around the world in the ‘80s, deep vein coagulation started to be reported in leprosy patients treated with rifampin, the main mycobactericidal drug of the multidrug therapy, and thalidomide, the anti-inflammatory drug of choice to treat ENL [16, 17]. For that reason ENL patient’s are the most susceptible ones to develop coagulation disorders due to the ENL pathophysiology *per se*, as well as the concomitant use of rifampin and thalidomide.

In a retrospective study of a cohort of 638 leprosy patients, we identified 35 patients who presented an atypical clot formation during sera harvesting. Until now, there have been no efforts to describe the composition or physiological explanation of this material, which has been identified by the medical routine as a lipid precipitation at the top of the blood clot that invades the serum fraction, here named as leprosum clot.

This study aimed to describe, for the first time, the occurrence of coagulation cascade exacerbation in some of these patients, as well as the protein profile of the leprosum clot, an abnormal lipid enriched clot which correlates with serum prothrombotic markers.

## Materials, subjects and methods

### Patients and samples

The present work was developed through the analysis of two groups of patients: a prospective group, which plasma samples were collected before multidrug therapy against leprosy, composed of 11 non-reactional (multibacillary leprosy; MB-NR), being 10lepromatous leprosy (LL) and 1 borderline lepromatous (BL). The erythema nodosum leprosum patients group (MB-ENL) included 13 LL and 1 BL individuals. These two groups include 6 female, 19 male with median age of 45.2 years, ranging from 23 to 80 (S1 Table). All samples were harvested before starting treatment, adopting the following criteria for exclusion: pregnancy, recent vaccination, presence of co-infections, autoimmune, and/or allergic diseases. A retrospective cohort were composed of 638 leprosy outpatients at the Souza Araújo Outpatient Unit of Oswaldo Cruz Institute, Fiocruz, from 2012 to 2014, where 35 patients presented the leprosum clot during serum harvesting. Samples were collected along multidrug therapy against leprosy, separated in: before and during the leprosum clot occurrence. Among these leprosum clot positive patients 48% were diagnosed with multibacillary leprosy (13 female, 22 male; medium age, 42.3 years; range 10–76), and 45,7% developing some reactional episode (4 patients suffering type 1 and 12 patients developing type 2) (S2 Table).

Blood serum and plasma samples were harvested using BD Holder (adaptor) and sterile one-use needles. For serum samples, ten milliliters of venous blood was collected in sterile BD Vacutainer SSTII Advanced tubes from all studied individuals, without additive or clot activator. The tube was centrifuged for 15 minutes at 800 x g in room temperature without brake. After this step, the supernatant fluid (serum; ±4 mL in normal samples, ±1 mL in samples where leprosum clot occurred) was collected with sterile serological pipete, aliquoted into cryovials (500μL/vial) and stored at -20°C until use. Plasma was also harvested from venous blood, collected from all volunteers with 5 mL vacutainer tubes, containing ~1.8 mg K2EDTA/mL blood. The tube was gently inverted for 10 times to mix blood and anticoagulant and the sample was centrifuged immediately for 10 minutes at 600 x g at room temperature. The supernatant (plasma) was carefully aspirated with sterile serological pipete, aliquoted into cryovials and store at -20°C until use.

The leprosy patients followed routine examinations and were classified according to the Ridley and Jopling criteria [18]. The multibacillary/pauciballary proportion and occurrence of reactional episodes were described. In addition, fibrinolysis/coagulation parameters were determined in 50 non-leprosy patients (23 male, 27 females; median age, 48 years; range: 28–66).

### SDS-PAGE, 2D electrophoresis and protein mass spectrometry

To establish the proteomic profile of the control and leprosum clot, leprosum clots were collected and immediately frozen at -70°C when occurred during patients serum harvesting. For comparison purposes, we generate control clots as followed: plasma samples from 6 healthy donors (3 male, 3 females; median age, 40 years; range: 26–60) were collected as described before. Immediately after, 5ml of each plasma sample was clotted by the addition of calcium chloride (0.5M). Control and leprosum clots were transferred using sterilized tweezers to glass tubes where proteins were extracted by maceration in extraction solution (7 M urea, 2 M thiourea, 4% CHAPS, 40 mM Tris and 60 mM DTT) followed by 5 freeze-thaw cycles. Protein content was measured with the commercially available 2D Quant-Kit (GE Healthcare, Ohio, USA) according to the manufacturer’s instructions. For SDS-PAGE analysis, 30 μg of leprosum clot and control clot protein extracts were solubilized in sample buffer (1.25 ml of pH 6.8 Tris to 0.5 M, 4 ml of glycerol, 0.2 g of SDS, 0.5 ml of β-mercaptoethanol, 0.25 ml of bromophenol blue and 0.05% deionized water) and applied in a 10% polyacrylamide gel with a 4% stacking gel. The electrical conditions employed were 30 min at 10 mA/gel and 20 mA/gel. For 2D electrophoresis, 500 μg of protein extract was subjected to isoelectric focusing using 24-cm IPG strips with a linear pH range between 4 and 7 [19]. The samples were solubilized in rehydration solution (7 M urea, 2 M thiourea, 4% w/v CHAPS, 0.002% w/v bromophenol blue, 60 mM DTT and 1% v/v IPG buffer of pH 3–10 or 4–7) and applied to the IPG strips. The electrical conditions used in the first dimension in the Ettan IPGphor system (GE Healthcare, Ohio, USA) were 30 volts for 12 h at 20°C for strip rehydration, followed by 200 volts for 1 h, 500 volts for 1 h, 1000 volts for 1 h, 1000–3500 volts for 0.5 h and 3500 volts for 4 h. After isoelectric focusing, the strips were incubated for 15 min with agitation in 10 ml of equilibrium solution (1.5 M of pH 8.8 Tris-HCl, 6 M urea, 30% v/v glycerol, 2% w/v SDS w/v, 0.002% w/v bromophenol blue) containing 100 mg of DTT, which was then replaced by another equilibrium solution containing 400 mg of iodoacetamide. Then, the strips were placed on a polyacrylamide gel (12%) with SDS [20], and the system was sealed with 0.5% w/v agarose at 80°C in Tris-glycine electrode buffer. The electrical conditions were 5 mA/gel for 30 min, followed by a constant 10 mA/gel until the end of the run. The gels were stained with ammoniacal silver for visual detection or colloidal Coomassie for identification by mass spectrometry. The spots were taken from the gel, digested with trypsin and analyzed by spectrometry MALDI-TOF/TOF 5800 (AB SCIEX, Clotachusetts, USA). The mass spectrometry protein identifications were obtained with a 5800 Proteomics Analyzer (Applied Biosystems, Foster City, CA). Both MS and MS/MS data were acquired in positive and reflectron mode using a neodymium-doped yttrium aluminum garnet (Nd:YAG) laser with a 200-Hz repetition rate. Typically, 1,600 shots were accumulated for spectra in the MS mode, whereas 3,000 shots were accumulated for spectra in the MS/MS mode. Up to ten of the most intense ion signals with a signal-to-noise ratio greater than 20 were selected as the precursors for MS/MS. External calibration in MS mode was performed using a mixture of four peptides: des-Arg1-Bradykinin (m/z = 904.47), angiotensin I (m/z = 1,296.69), Glu1-fibrinopeptide B (m/z = 1,570.68) and ACTH (18–39) (m/z = 2,465.20). MS/MS spectra were externally calibrated using known fragment ion masses observed in the MS/MS spectrum of Glu1-fibrinopeptide B. MS/MS database searching was performed against the NCBInr databases using the Mascot software (www.matrixscience.com). The search parameters included two missed tryptic cleavages allowed and non-fixed modifications of methionine (oxidation) and fixed cysteine (carbamidomethylation). The peptide per sample plate (pps) and peptide per well (ppw) files were generated from the raw (or native) MS data according to the following parameters using the Data Explorer Software (Applied Biosystems). The parameters for MS1 were as follows: mass range, 900–3,500 Da; peak density, 200 peaks per 200 Da; signal-to-noise ratio 30; minimum area 1000 μm^2^; and maximum peaks per spot 60. The parameters for MS2 were as follows: mass range 60 Da until the mass of the precursor; peak density 55; 200 peaks per 200 Da; signal-to-noise ratio 2; minimum area 10 μm^2^; maximum peaks per precursor 60. Protein identifications based MS/MS peptide were validated in Scaffold 2 software (Proteome Software Inc., Portland, OR), and the identifications were accepted if they could be established at greater than 95% probability, as specified by the Peptide Prophet algorithm [21], and contained at least 2 identified peptides. Protein probabilities were assigned using the Protein Prophet algorithm [22].

### Protein visualization and image analysis

Gels were stained with colloidal Coomassie Brilliant Blue G-250 and documented using a GS-800 auto-calibrating imaging densitometer (Bio-Rad). Image analysis was performed using PDQuest software, version 8.0.1 (Bio-Rad). Comparative 2D data were derived from three biological replicates from each clot type (control clot and leprosy clot). The spots were quantified based on their relative ‘volume’: the amount of a protein spot was expressed as the sum of the intensities of all pixels composing that spot. To compensate for subtle differences in sample loading, gel staining and de-staining, the volume of each spot was normalized relative to the total density of valid spots present in the gel image. After automated detection and matching, manual editing was conducted.

### Protein identification

Following MS acquisition, each spectrum was submitted to a peptide mass fingerprinting search for MS/MS spectra using Mascot version 2.5 (Matrix Science: http://www.matrixscience.com/). For protein identification, the search was performed against the NCBI-nr nonredundant database (NCBI-nr201512, National Center for Biotechnology Information, http://www.ncbi.nlm.nih.gov/) taxonomy restricted to Homo sapiens. For Mascot searches, the parameters used were trypsin as the enzyme of choice and two missed cleavage, ±50 ppm peptide tolerance, and ±0.6 Da for the fragment ion mass (MS/MS tolerance). Oxidation of methionines was allowed as variable modification, whereas alkylation of cysteines (carbamidomethyl cysteines) was set as constant modification. Identification was considered valid for Mascot protein scores greater than 50 and a significance threshold of p<0.05. If a protein ‘hit’ was identified by only one peptide, the MS/MS data were required to exhibit a clear spectrum with sequence tags that matched at least three consecutive y or b fragment ion series. Lastly, a good correlation between the experimental and theoretical molecular mass and pI was also considered for positive identifications. A functional protein association network, STRING (http://www.string-db.org), was used for interaction networks. The search was based in the UniProt name, and Homo sapiens was the selected organism.

### Lipid extraction and analysis

The leprosum and control clots were macerated, and lipids were extracted with chloroform, methanol, and water (1:2:0.8, v/v/v) as described previously [23]. Neutral lipids and phospholipids were analyzed through one-dimensional HPTLC on silica gel 60 plates (Merck, Darmstadt, Germany). For neutral lipids analysis, the plates were first developed on hexane-ethyl ether-acetic acid (60:40:1, v/v/v) until the solvent border reached the middle of the plate and then on hexane-chloroform-acetic acid (80:20:1, v/v/v). For phospholipids analysis, the plates were developed on chloroform-methanol-acetone-acetic acid-water (40:13:15:12:8). HPTLC plates were stained by spraying with a charring solution consisting of 10% CuSO_4_ and 8% H_3_PO_4_ and then heating to 180 °C for 5–10 min as described previously [24]. The charred TLC plates were then subjected to densitometric analysis using ImageJ software. The percentage of each lipid was calculated from the total amount of lipid (set as 100%) isolated in each clot.

### *M*. *leprae in vitro* infection

Live *Mycobacterium leprae*Thai-53 strain was prepared from athymic nu/nu mouse footpads immediately before use and was provided by Dr. Patricia Sammarco Rosa (Lauro de Souza Lima Institute, Department of Biology, Bauru-SP, Brazil). *M*. *leprae* preparation, viability determination and purity were performed as described elsewhere [25]. HEPG-2 human hepatocyte cell lineages were obtained from American Type Culture Collection (ATCC) and maintained in high-glucose D-MEM (LCG Bioscience, São Paulo, Brazil) supplemented with 10% fetal bovine serum (CULTILAB, Campinas, Brazil) without antibiotics. Cultures were kept at 37°C in a humidified 5% CO2 atmosphere. Infection was performed over 48 h, with a multiplicity of infection of 50 *M*. *leprae* per cel at 33°C in a humidified 5% CO2 atmosphere. Complement component 4 (C4) and inter-alpha-trypsin inhibitor protein (IHRP) release were measured in the supernatant as described below.

### Immunoassays and coagulation parameters analysis

In order to avoid rifampicin and thalidomide interference of in our data, aPPT, PT, d-dimer and fibrinogen parameters were determined in all patients before treatment. In the present study we applied Stago’s STA-R Evolution instrument (Stago, Asnièressur Seine, France) to determine partial thromboplastin time (aPPT) and prothrombin time (PT) in all plasma samples, according the manufacturer instructions. The levels of von Willebrand and soluble tissue factor, C4 complement, and anti-cardiolipin IgM antibody in the serum of leprosy patients were determined using the following commercial kits: Human von Willebrand Factor ELISA kit and Human Tissue Factor ELISA kit (Abcam, Clotachusetts, US) and C4 turbiquest (Labtest, Minas Gerais, Brazil), respectively. HDL-cholesterol, total cholesterol and triglycerides were determined through high-throughput enzymatic colorimetric test Cobas 8000 module 702 (Roche, Pleasanton, USA). When triglyceride levels were less than 400 mg/dL, cholesterol was determined using the Friedwald formula. Fibrinogen levels were determined using automatic Clauss methodology with a BCS high-throughput reader (Siemens, Berlin, Germany). D-dimers were determined using imunoturbidimetric methodology with a BCS reader (Siemens, Berlin, Germany). All tests were performed according to the manufacturer’s instructions. For quantification, the absorbances of samples were compared with the standards using a mathematical correlation by linear regression of the standard curve data.

### Statistical analysis

All numerical data were analyzed using non parametric tests, Kruskall-Wallis with post-test to compare relevant groups, or Mann Whitney test to compare continuous variables. Fisher Test was used to compare categorical data, with GraphPad Prism software.

### Ethics statement

This project was approved by the Oswaldo Cruz Foundation Research Ethics Committee (protocol number 275.648) and all methods were performed in accordance with the Brazilian Guidelines and Norms for Research Involving Human Beings (CNS 466/2012), and to the principles expressed in the Declaration of Helsinki. Informed written consent was obtained from all individuals prior to specimen collection. Parents and guardians provided consent on behalf of all patients below 18 years.

## Results

**Fig 1:**
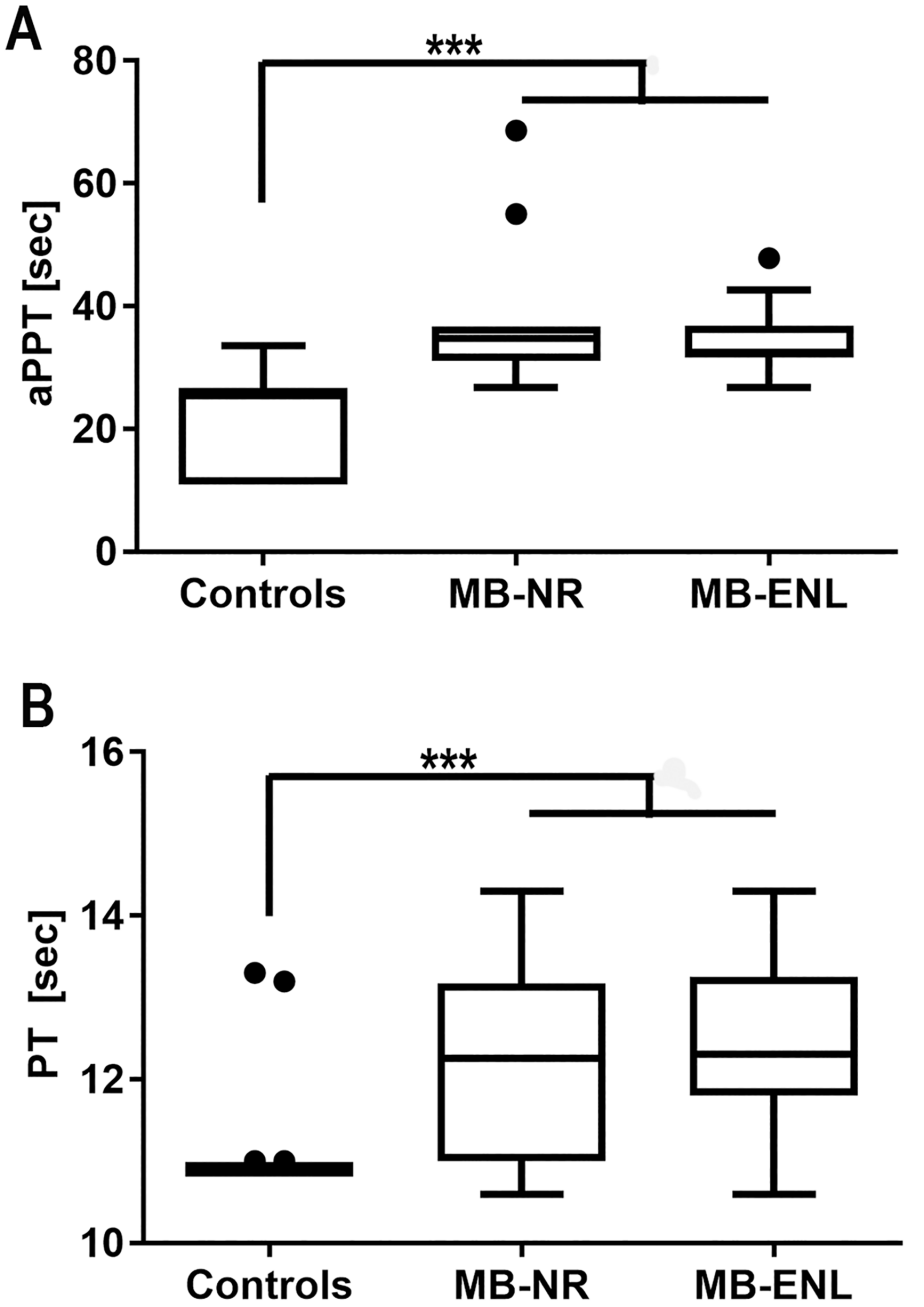
Multibacillary leprosy patients present prolonged coagulation time in both coagulation pathways. Coagulation times were determined on plasma from 50 non-leprosy patients (Controls), 9 non-reactional multibacillary patients (MB-NR) and 15 multibacillary erythema nodosum leprosum patients (MB-ENL). A) Activated partial thromboplastin time test (aPPT); B) prothrombin time test (PT). MB-NR group are composed by 10LL and 1 BL patients. MB-ENL group are composed by 13 LL and 1 BL individuals. Neither of them presented the leprosum clot during serum harvesting. All patients’ details are listed in S1 Table. *** indicate p<0.0001 with ANOVA.

To evaluate the hemostatic parameters among leprosy patients, we prospectively analyzed plasma samples from non-leprosy patients and multibacillary (MB) patients developing or not a type 2 reaction (ENL) (S1 Table). During this prospective phase of the study, in a serum harvesting performed in parallel, none of the patients developed the leprosum clot. We first investigated the contact activation and tissue factor coagulation pathway efficiency by measuring the activated partial thromboplastin (aPTT) and prothrombin time (PT), respectively (Fig 1A and 1B). Both MB patient groups exhibited significantly prolonged aPTT and PT times, indicating dysfunction or intravascular consumption of one or more factors involved in both coagulation pathways.

**Fig 2:**
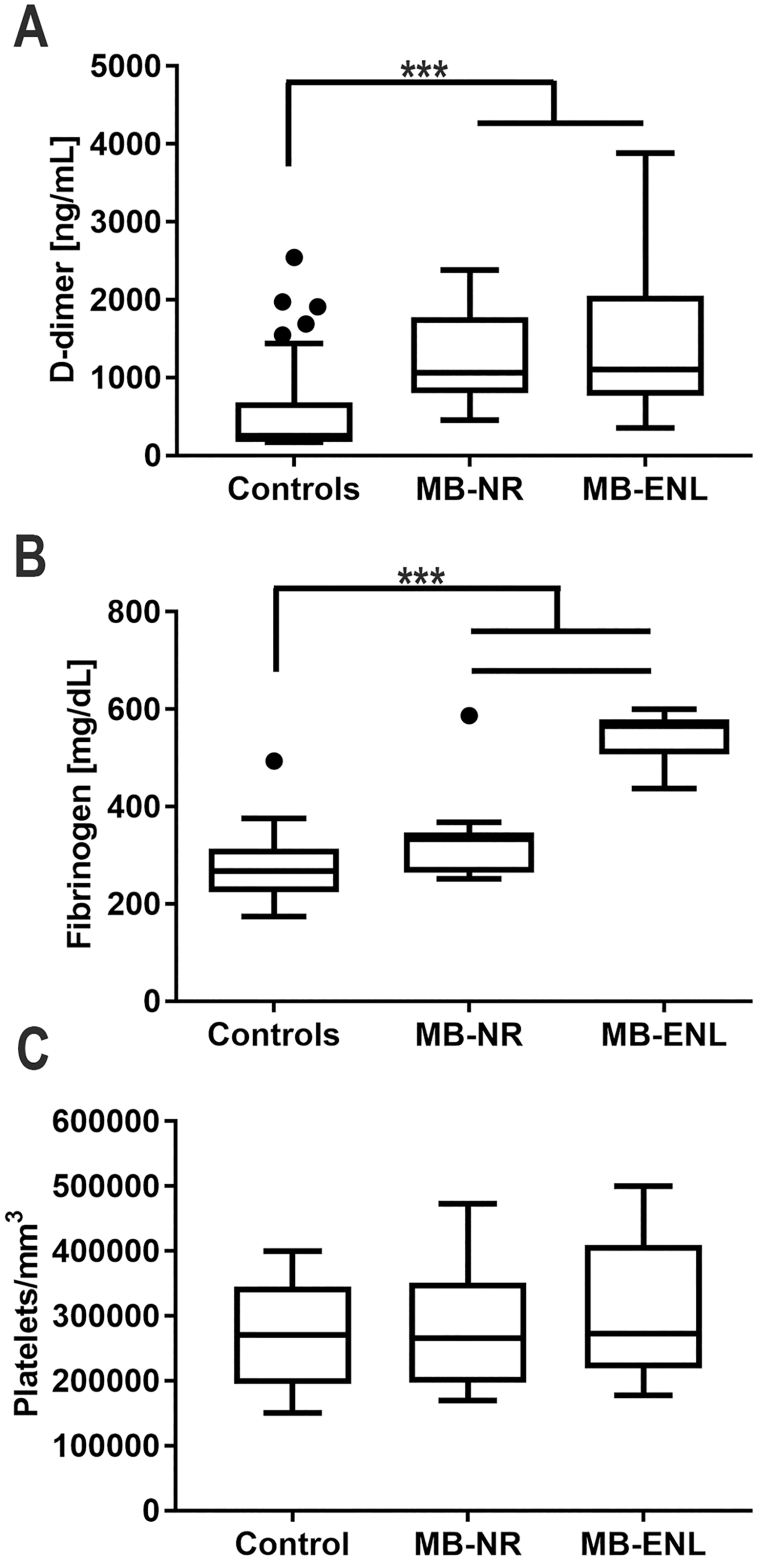
Multibacillary leprosy patients present a procoagulant phenotype. Coagulation parameters were determined on plasma from 50 non-leprosy patients (Controls), 9 non-reactional multibacillary leprosy patients (MB-NR) and 15 multibacillary erythema nodosum leprosum patients (MB-ENL). A) Fibrinogen and B) d-dimer plasma concentrations were determined. C) Platelet counting showed no alteration among samples. MB-NR group are composed by 10LL and 1 BL patients. MB-ENL group are composed by 13 LL and 1 BL individuals. * indicate p<0.01 and *** indicate p<0.0001 with ANOVA.

Next, we determined the d-dimer levels in leprosy MB patients, demonstrating that it is significantly increased in both MB patient groups. Since high d-dimer levels indicate that both thrombin and plasmin generation have occurred, this data suggests increase of intravascular coagulation and fibrinolysis in MB patients (Fig 2A). In contrast, fibrinogen, an acute phase reactant, is increased in ENL patients, as expected (Fig 2B). Interestingly, the prothrombotic state of leprosy patients had a minor impact on platelet counts (Fig 2C), thus ruling out the occurrence of disseminated intravascular coagulation in these patients.

**Fig 3:**
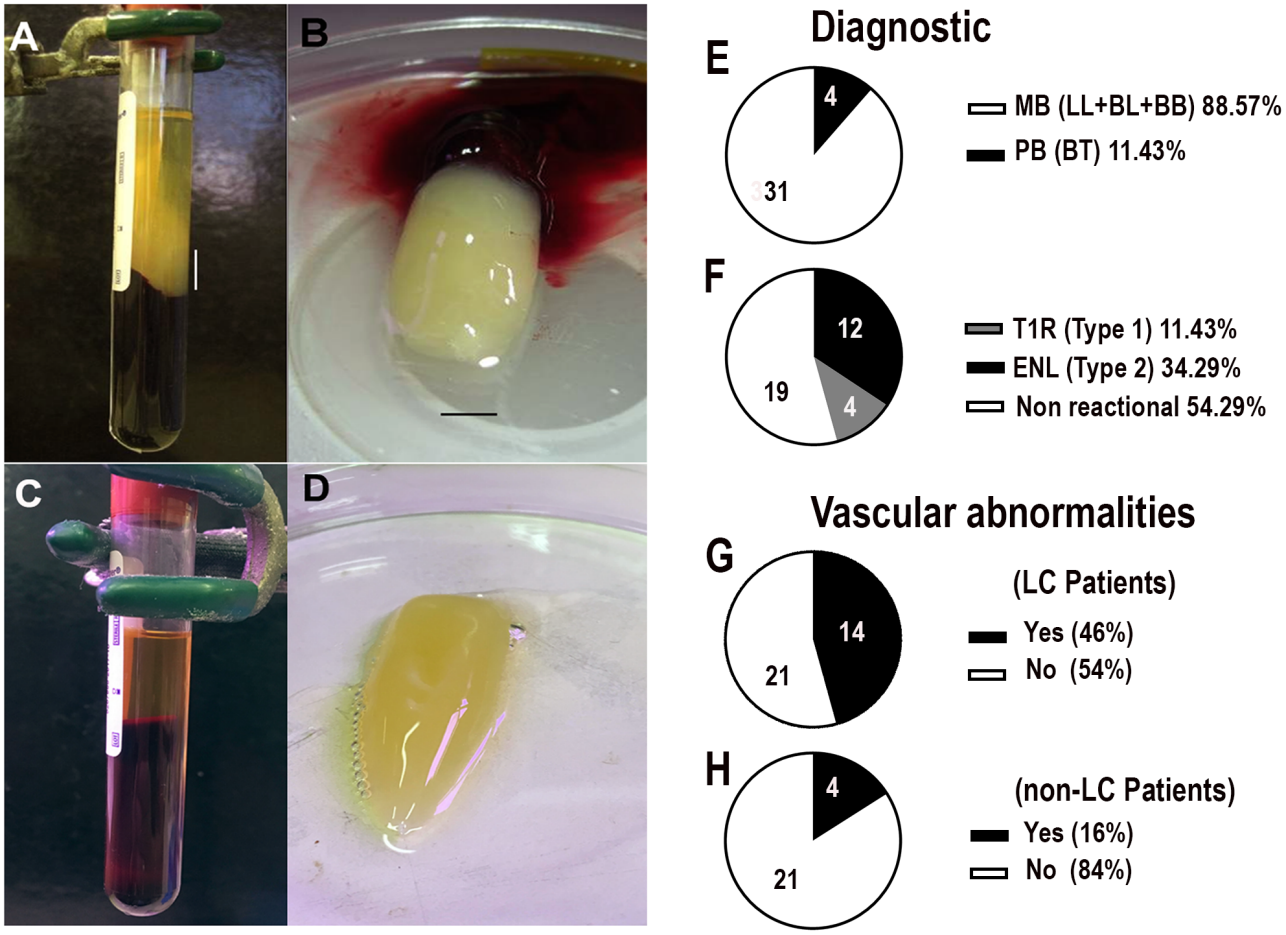
Leprosum clots formed during serum harvesting of multibacillary patient. A) Appearance of the leprosum clot during lepromatous patient serum harvesting, after blood coagulation and subsequent centrifugation. The vertical scale bar represents 2 cm and identifies the leprosum clot appearing on the top of normal erythrocyte-rich clot. B) The leprosum clot after PBS washing had a characteristic white-milky color. Scale bar represents 1 cm. C) Representation of a normal blood serum harvesting, after coagulation and subsequent centrifugation. D) Control clot aspect, formed from healthy donors’ plasma by the addition of calcium chloride; E) Leprosum clot distribution among 35 leprosy outpatients (11 females, 24 males; median age, 47.7 years; range, 10–76 years) discriminated by the clinical pole of the disease or presence/absence of reactional episodes (F). G) Vascular abnormalities occurrence in patients who developed leprosum clot during serum harvesting (LC patients) or not (H). MB: multibacillary patients, PB: paucibacillary patients, ENL: erythema nodosum leprosum and T1R: reversal reaction.

In a second approach, we retrospectively analyzed the clinical history of 638 leprosy patients treated at the Souza Araujo Outpatient Unit. Interestingly, during blood harvesting, 35 patients presented a milky white mass that had formed on the top of the blood clot during serum harvesting. Herein, this material is referred to as “leprosum clot” (Fig 3). Among these patients, 48% were diagnosed with multibacillary leprosy and 45,7% developing some reactional episode (S2 Table). Therefore, our data indicated that formation of the leprosum clot is a rare event, commonly associated with the MB pole of the disease (p<0.0001) (Fig 3E). We also observed the occurrence of vascular abnormalities, such as upper and lower limbs cyanosis, edema and ulcerations, more frequently observed in the cohort of patients who formed the leprosum clot during serum harvesting (Fig 3G and 3H, S1 and S2 Tables).

**Fig 4:**
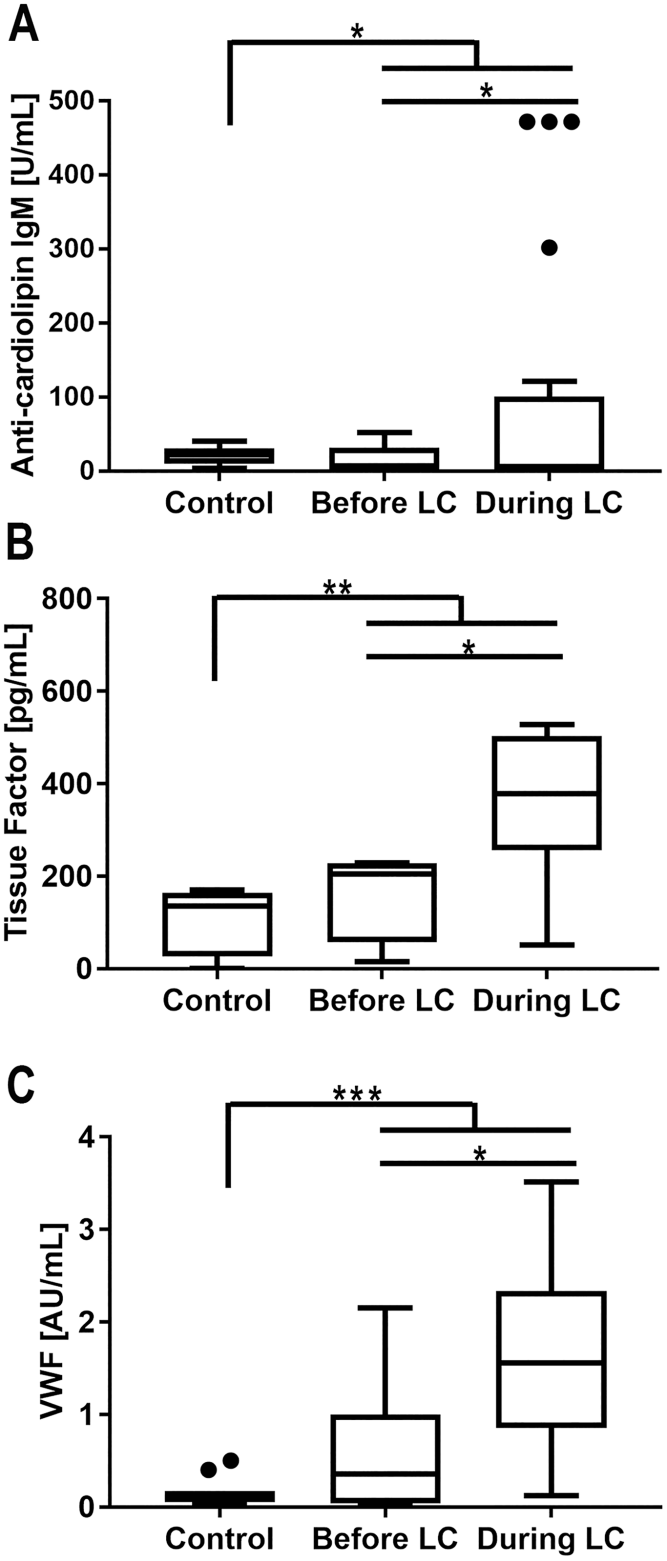
Correlation between the leprosum clot and pro-coagulant factors. Pro-coagulant factors were determined in multibacillary leprosy patient serum presenting the leprosum clot (during LC). We also performed the same analysis in previously harvested serum, varying from weeks to months, from the same group of patients before the occurrence of the leprosum clot (before LC). Levels of anti-cardiolipin IgM (A), soluble tissue factor (B) and von Willebrand factor (C) were determined in serum from 35 leprosy outpatients (11 female, 24 male; mean age, 48 years; range, 10–71 years). 1AU indicates the 1 μg/mL signal of affinity chromatography purified anti-cardiolipin IgM. * indicates p<0.01, ** indicates p<0.005 and *** indicates p<0.001 with ANOVA.

Following WHO multidrug therapy, we collected serum samples of all patients at three times: before treatment, during treatment when some intercurrence such as ENL occurs, and after treatment. Among these 35 patients listed in S2 Table, leprosum clot occurred randomly, before, during or after treatment, only once per patient. We were able to assess frozen serum samples collected from patients which were negative for leprosum clot formation before treatment and during leprosy treatment developed the leprosum clot, observed in a subsequent blood harvesting. In some cases the leprosum clot was observed before treatment, and none of the individuals presented the leprosum clot after 12 doses of multidrug therapy. Unfortunately, functional analysis such as aPPT, PT and D-dimers, are not possible to be performed in such sample. On the other hand, we successfully observed that levels of anti-cardiolipin IgM, von Willebrand factor (vWF) and soluble tissue factor (TF) in the leprosum clot positive samples were moderately elevated compared with control sera (Fig 4). In contrast, these factors were dramatically elevated in the samples which formed the leprosum clot during harvesting. Therefore, leprosum clot formation appears to be directly correlated with increased levels of factors associated with prothrombotic states.

Through the complementation of calcium in plasma samples from healthy donors, we generated control clots, determining the lipid profile of both clots by high-performance thin layer chromatography (HPTLC) analysis. Control and leprosum clots exhibited a distinct macroscopic patterns; the leprosum clot was white in color and had a solid appearance, whereas a control clot had characteristic transparent, gelatinous and delicate appearance.

**Fig 5:**
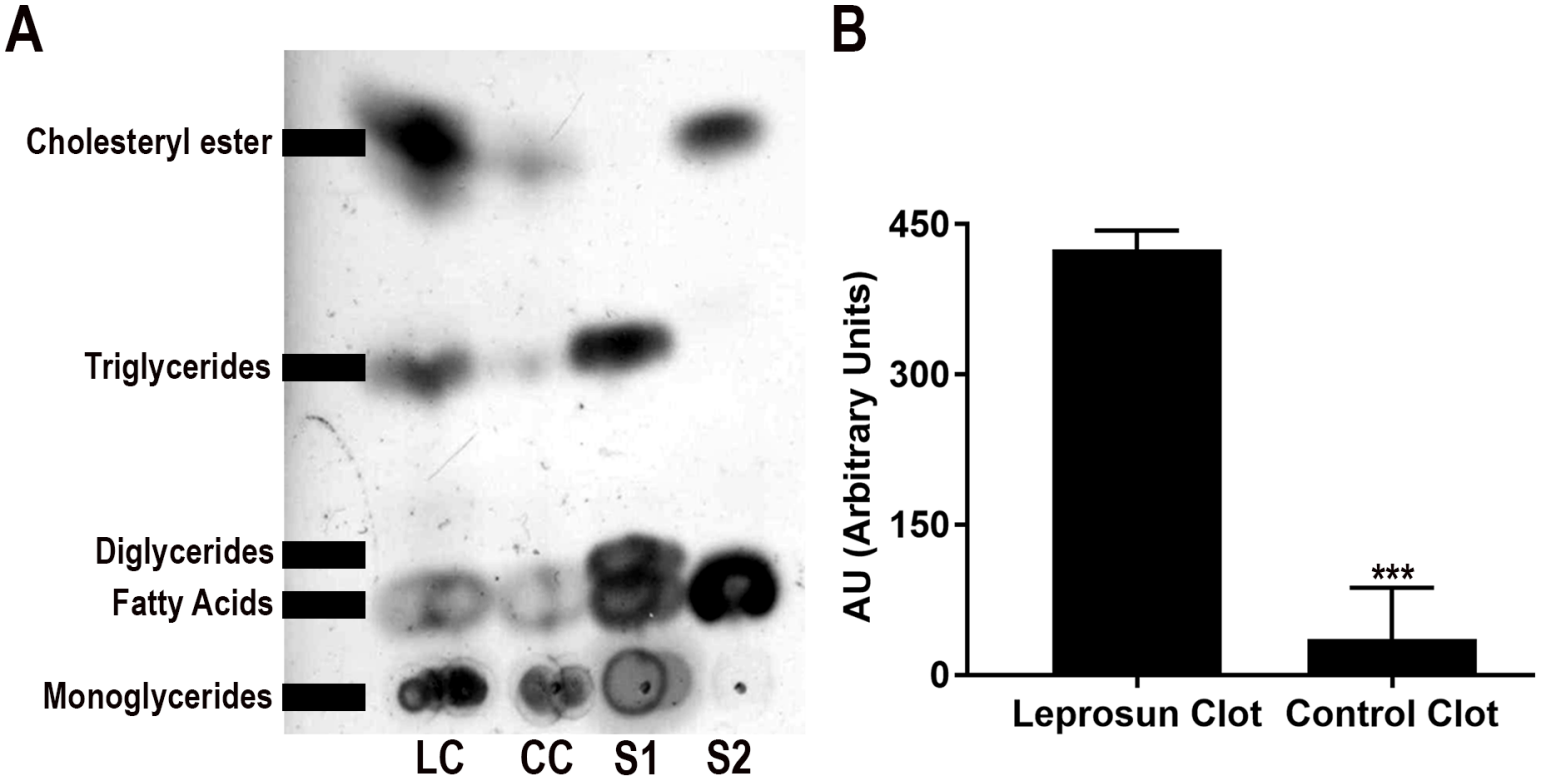
Neutral lipid content in control and leprosum clots. A) Representative image of a high-performance thin layer chromatography of a leprosum clot (LC) and control clot (CC) showing similarities in their compositions but differences in their amounts of neutral lipids. The lipid standards, S1 and S2, were composed of monoglycerides, diglycerides, and triglycerides (S1) and fatty acids and cholesteryl ester (S2). B) Densitometric analysis of (A) by ImageJ software shows the relative abundance of total neutral lipids in each sample in arbitrary units. Image is representative of four independent experiments. *** indicates p<0.0001 by t test using Mann-Whitney.

By comparing neutral lipids amounts from leprosum and control clots, we demonstrated that leprosum clots present approximately ten-fold more cholesteryl esters and triglycerides than control clots (Fig 5A and 5B), explaining the visual differences between the clots. These data corroborate the intuitive conclusion of physicians and nurses, which erroneously recognized the leprosum clot as a lipid mass. Interestingly, multibacilary leprosy patients present low levels of total and HDL-related cholesterol in their sera, while other related sera parameters such as triglycerides, LDL and VLDL-related cholesterol were normal (S2 Fig).

**Fig 6:**
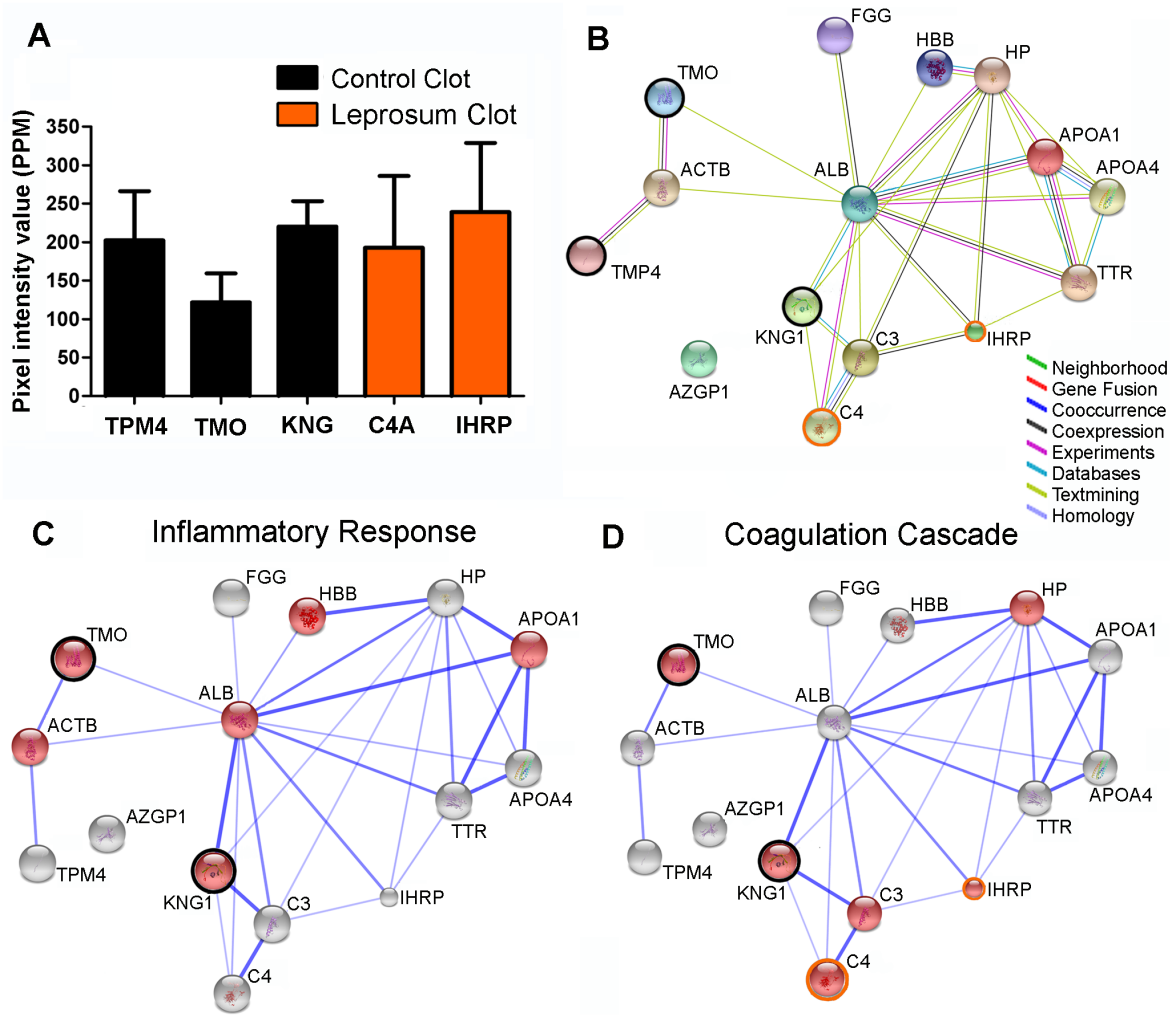
Protein-protein interaction network constructed by STRING 10. (A) Protein differential identification by 2DE in control (CC) and leprosy clots (LC). Pixel intensity value in PPM for each protein. Black bars represent proteins exclusively identified in CC: TPM4 (tropomyosin alpha-4), TMO (tyrosine 3-monooxygenase) KNG1 (kininogen 1), and orange bars represent proteins identified only in LC: C4A (complement component 4A) and IHRP (inter-alpha-trypsin inhibitor family heavy chain-related protein). (C) Confidence view based on the Gene Ontology enrichment to biological processes related to blood coagulation (p = 1.469^−3^); (B) Evidence view showing all detected proteins in both clots by different line colors representing the types of association evidence (only associations with p = 1.98^−13^ are shown); (C) and inflammatory response (p = 6.369^−4^). Stronger associations are represented by thicker lines. IHRP is represented by a small sphere, indicating that there is no crystallographic data about its structure. Proteins highlighted by black rings were exclusively detected in control clots, and proteins highlighted by orange rings were exclusively detected in leprosy clots.

To understand the differences between leprosum and control clot proteomic profiles, we performed a comparative 2D gel electrophoresis proteomic analysis of three biological replicates from each clot type (S1 Fig). We extracted approximately 137 spots from each gel and identified these spots using MALDI-TOF analysis. Most spots were identified as fibrin (alpha, beta and gamma chains). We also identified apolipropotein A1, a hallmark of the high-density lipoprotein (HDL) fraction, among other serum proteins. Table 1 listed the 15 most abundant proteins found in the leprosum clot. After analysis using PDQuest software, protein spots exclusively presented in one type of clot were identified (Fig 6A). Using the STRING interaction network analysis, we constructed an interactome of these proteins based on different types of evidence (Fig 6B). From this set of proteins, three were only identified in the control clot: tropomyosin alpha-4 chain isoform Tpm4 (TPM4), tyrosine 3-monooxygenase/tryptophan 5-monooxygenase (TMO) and kininogen 1 (KNG1). The proteins inter-alpha-trypsin inhibitor family heavy chain-related protein (IHRP) and complement component 4A (C4A) were only identified in leprosum clot (Fig 6B). A graph theoretic network analysis revealed that six of these 15 proteins are involved in important biological processes, such as blood coagulation and inflammatory response (Fig 6C and 6D).

**Table 1:** Most abundant proteins identified on the 2DE gels exclusively in Control Clot (CC), Leprosy Clot (LC) or both (CC/LC) by MS/MS.

Protein name	Gene name	Uniprot*	Score	Theor.M (Da)	Theor. PI	Coverage (%)	Peptides identified (Unique peptides)	Peptides matches**	Sample
Actin beta	ACTB	P60709	566	42052	5.29	23	6(5)	6(6)	CC/LC
Apolipoprotein A4	APOA4	P06727	608	45353	5.33	21	8(8)	8(8)	CC/LC
Apolipoprotein A1	APOA1	P02647	592	28061	5.27	25	7(7)	7(7)	CC/LC
Complement component C3	C3	P01024	439	188585	6.02	4	6(5)	6(6)	CC/LC
Complement component C4	C4	P0C0L4	246	194337	6.65	2	4(4)	4(4)	LC
Fibrinogen gamma chain	FGG	P02679	555	50077	5.61	17	6(6)	6(6)	CC/LC
Haptoglobin	HP	P00738	549	38209	6.14	23	7(7)	7(7)	CC/LC
hemoglobin beta	HBB	P68871	115	16112	6.75	40	5(2)	5(5)	CC/LC
Inter-alpha-trypsin inhibitor family heavy chain-related protein (IHRP)	ITIH4	Q14624	295	103549	6.51	4	4(4)	4(4)	LC
Kininogen 1	KNG1	P01042	294	47871	6.29	9	6(6)	6(6)	CC
Serum albumin	ALB	P02768	780	71316	6.05	15	8(8)	8(8)	CC/LC
Transthyretin	TTR	P02766	626	15062	5.52	39	6(2)	6(6)	CC/LC
Tropomyosin alpha-4 chain	TPM4	P67936	411	28504	4.67	14	5(5)	5(5)	CC
Tyrosine 3-monooxygenase (TMO)	YWHAZ	P63104	55	27899	4.73	5	1(1)	1(1)	CC
Zn-alpha2-glycoprotein	AZGP1	P25311	171	34942	5.71	13	4(3)	4(4)	CC/LC

*http://www.uniprot.org/uniport

**Indicate peptides with individual ions scores > 38, i.e, peptides with identity or extensive homology (p<0.05) based on http://www.matrixscience.com/search_form_select.html

**Fig 7:**
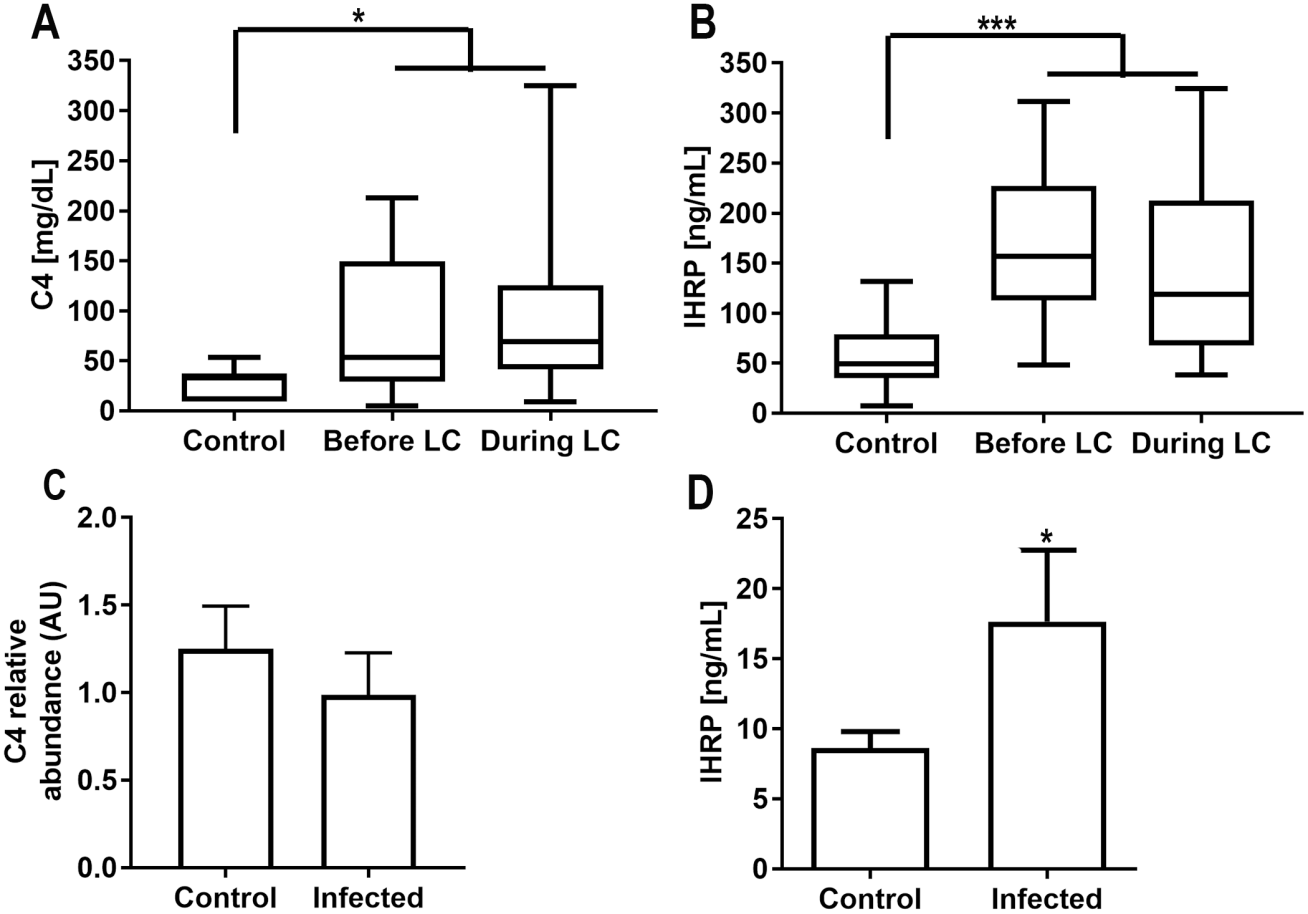
Complement component 4 (C4) and inter-alpha-trypsin inhibitor protein (IHRP) production are induced by *M*. *leprae*. IHRP and C4 were determined in multibacillary leprosy patient serum presenting the leprosum clot (during LC). We also performed the same analysis in previously harvested serum, varying from weeks to months, from the same group of patients before the occurrence of the leprosum clot (before LC). IHRP (A) and C4 (B) are more abundant in leprosy patient sera than healthy controls. Although *M*. *leprae* fail to induce C4 synthesis and release by human hepatocytes *in vitro* (C); we successfully mimetized the phenomenon *in vitro* observing strong IHRP induction in our model (D). * indicates p<0.05, and ** indicates p<0.001.

To validate these identifications, we performed an ELISA in order to quantify C4 and IHRP proteins in leprosy patients’ sera samples prior to and during leprosy clot occurrence (Fig 7A and 7B). We observed that both proteins are increased during and even before leprosum clot formation.

In order to mimics this phenomenon *in vitro*, we exposed HEPG2, a cell line derived from hepatocytes, the maintainer of these proteins blood levels [26, 27], to live *M*. *leprae*. As results we observed that *M*. *leprae* where able to induce IHRP synthesis and exportation to the medium after 48 h of infection. The same was not observed to C4 component, which probably is not directly related to *M*. *leprae* infection, but immune stimulation of hepatocytes (Fig 7C and 7D), or macrophages.

## Discussion

Leprosy persists as a significant public health problem worldwide. Despite the drastic reduction in the number of cases in recent years, new cases continue to be detected [28]. Procoagulant disorders in untreated leprosy patients were described along decades associated [6, 7] or not [11, 12, 13] to reactional episodes.

The immune response to infections by pathogens, such as bacteria and fungi, can be accompanied by changes in key metabolic pathways, which may include coagulation and lipid metabolism [29, 30]. In addition, expression of the clotting initiator protein, tissue factor, by blood immune cells plays a central role in the immunothrombosis, promoting microvessel thrombosis to capture microbes, thus limiting pathogen dissemination [31].

Hepatic infection by *M*. *leprae* was extensively reported in the past [32, 33]. Based on these data, we believe that the presence of high loads of *M*. *leprae* antigens in the blood stream of MB patients, and consequently in their liver, as well as the hepatic modulation by immune system in response to these antigens, could be responsible to induces acute phase protein synthesis with a high thrombogenic effect. For this reason, the exacerbation of fibrin clot generation observed in our patients could be attributed to the hepatic increment in expression of fibrinogen, von Willebrand factor and tissue factor, that are likely associated with pro-inflammatory cytokines previously described in ENL [34, 35].

The literature described that leprosy patients developing erythema nodosum leprosum (ENL) present prolonged activated partial thromboplastin time (aPTT), normal prothrombin time (PT) and high fibrinogen and platelet titers [7]. Our data, in contrast, showed both coagulation time parameters prolonged in leprosy patients’ plasma, which indicates a disturbance not only in the tissue factor pathway, but also in the contact activation pathway. We suggested that these data discrepancy could be attributed to differences between methods, populations and patient cohorts.

Fibrinogen, abundance and cleavage, were also increased in these patients, as demonstrated by the high levels of d-dimers. Because the association of fibrin-von Willebrand [36] and von Willebrand-apolipoprotein A1 [37] which has been previously described, we first hypothesized that the high levels of von Willebrand factor, followed by low levels of HDL in leprosy patients sera, could be explained by cross-linking of HDL to the fibrin, increasing its amount of neutral lipids, resulting in the leprosum clot. Unfortunately, we were not able to observe differences in apolipoprotein A1 abundance between control and leprosy clots in 2D gels, and because of this, the source of the neutral lipids contained in the leprosum clot remains a subject of investigation.

During sepsis treatment, exacerbation of soluble tissue factor serum levels and the resultant coagulation activation is a major cause for infection-associated mortality and inflammation[34]. It has been already described that those patients with antiphospholipid syndrome associated to high levels of blood tissue factor presents higher risk to develop thromboembolic complications[35]. Here, a correlation between high levels of soluble tissue factor, anti-cardiolipin IgM, increased von Willebrand factor, and leprosum clot occurrence was observed (Fig 4). We attribute this to the fact that mycobacterial cell wall components are able to induce a series of response in a large number of cells, as example, tissue factor expression in macrophages and endothelial cells [38].

*M*. *leprae* infection has been shown to induce antiphospholipid antibodies and complement factors, such as the membrane attack complex or MAC, that contribute to the peripheral nervous damage observed in leprosy [12, 39]. The complement system plays a major role in immune and inflammatory responses, comprising more than 30 proteins associated with cell membranes or found in plasma. The pathway activation results in the formation of anaphylatoxins (C3a, C4a, C5a) and MAC [40]. The differential proteomics analysis of the leprosum clot demonstrated the presence of anaphylatoxins (C4) and IHRP protein. These proteins are related but not directly involved in the leprosum clot formation, because high levels of both proteins were also observed in leprosy patients sera weeks prior to leprosum clot occurrence. It was already demonstrated that complement activation products are highly abundant in leprosy reactional episodes, and their presence in the leprosum clot is probably circumstantial [41]. IHRP, which is also increased in patient’s sera and blood clot, belongs to the inter-alpha-trypsin inhibitor family of acute phase proteins, comprising the common light chain bikunin. Its protease inhibitory activity is involved in suppression of TNF–α signaling in macrophages [42], as well as binding to polimorphonuclear cell surface actin, inhibiting their phagocytic activity [43], a process which could be involved in the well-known immune responsiveness against *M*. *leprae* during infection [44]. Bikunin high levels is also related to stabilization of fibroblasts extracellular matrix [45], a processes related to irreversible nerve damage in leprosy [46]. The consequences of IHRP increase in leprosy patient’s sera, as well as its induction in hepatocytes infected by *M*. *leprae in vitro* represent an interesting line of investigation. More important, the absence of kininogen 1 in the leprosum clot, which deficiency was already associated with prolonged aPPT [47], could be a reasonable explanation for the observation of prolonged aPPT in leprosy patients.

There are clinical symptoms, frequently observed in leprosy patients, which could be related to intravascular coagulation, such as limbs cyanosis and edema, as well as non-diabetic foot ulceration, related to the occlusion of superficial vessels. One possibility that cannot be ruled out is that this leprosy multibacilary patients procoagulant status could contribute to the disturbance in venous return dynamics observed in the ENL, responsible for the classic skin lesions and plaques shared by ENL and lipodermatosclerosis [48].

Taken together, our data strongly suggest that although the occurrence of leprosum clot represents a rare phenomenon associated with procoagulant exacerbation and vascular abnormalities, all leprosy MB patients enrolled in the present work develop a procoagulant status, presenting exacerbation of *in vitro* clot formation in some acute cases due to high levels of fibrinogen, anti-cardiolipin antibodies, von Willebrand factor and soluble tissue factor. We characterize the nature of leprosum clot, describing a panel of serum proteins which are up-regulated in multibacilary leprosy patients, such as D-dimers, anti-cardiolipin, von Willebrand factor, soluble tissue factor, C4 and IHRP. We also observed plasma fibrinogen level increased in patients developing leprosy reactional episodes. Monitor multibacilary patients fibrinogen levels in order to predict reactional episodes [35] could represent an advance in the treatment, avoiding ENL complications such as nerve and tissue damage. We propose that multibacilary patients with high levels of fibrinogen could be beneficiated from a prophylactic use of xanthine derivatives such as pentoxifylline, in order to prevent some of the acute clinical symptoms observed during severe cases of leprosy reactional episodes, such as cyanosis and tissue necrosis, probably related with superficial vein thrombosis [49, 50].

## Supporting information

S1 Table Identification of leprosy patients included in the prospective part of study. (DOCX)

S2 Table Identification of leprosy patients which presented the leprosum clot (LC+). (DOCX)

S1 Fig Protein patterns and similarities between leprosum and control clots. 2D gel differential analysis between a control and leprosum clot protein fractions on IPG strips covering pH 4–7. Representative gels stained with Coomassie colloidal blue were spots were trypsin-digested and identified by MALDI-TOF. Spots delimited by an orange box correspond to alpha fibrin; black box correspond to beta fibrin; green box correspond to gamma fibrin; purple box correspond to apolipoprotein A1 and red box correspond to beta actin. Proteins exclusively found in control or leprosum clot are identified with circles as follow: black circle correspond to kininogen 1, purple circle correspond to Tropomyosin alpha-4, blue circle correspond to Tyrosine 3-monooxygenase, orange circle correspond to IHRP and green circle correspond to Complement C4. Arrows indicate the direction of separation by isoelectric point (IEF) and molecular weight (MW).
(TIF)

S2 Fig Leprosy patient sera apolipoproteins and neutral lipids profile. Total plasma triglycerides (A), LDL-cholesterol (B) and VLDL-cholesterol (C) levels were determined for 50 non-leprosy patients plasma (controls), MB-NR group are composed by 10 LL and 1 BL patients. MB-ENL group are composed by 13 LL and 1 BL individuals. D) Total plasma cholesterol and HDL-cholesterol (E) determined for 50 non-leprosy patients plasma (controls), MB-NRgroup are composed by 10 LL and 1 BL patients. MB-ENL group are composed by 13 LL and 1 BL individuals.*** indicates p<0.0001 with ANOVA.
(TIF)
