# Supplementary material for: Blood coagulation abnormalities in multibacillary leprosy patients
Source: PLoS Negl Trop Dis. 2018 Mar 22;12(3):e0006214. doi: 10.1371/journal.pntd.0006214 (PMC5863944; doi:10.1371/journal.pntd.0006214)
Supplement: S1 Table — (DOCX) [file pntd.0006214.s001.docx]

**Supplementary Table 1 - Identification of leprosy patients included in the prospective part of study**

| ID | Age (years) | Form of disease | Reaction | Gender |
| --- | --- | --- | --- | --- |
| LL1 | 46 | LL | NR | M |
| LL2 | 80 | LL | NR | M |
| LL3 | 55 | LL | NR | M |
| LL4 | 35 | LL | NR | F |
| LL5 | 23 | LL | NR | F |
| LL6 | 32 | BL | NR | M |
| LL7 | 54 | LL | NR | F |
| LL8 | 50 | LL | NR | M |
| LL9 | 33 | LL | NR | M |
| LL10 | 52 | LL | NR | M |
| LL11 | 54 | LL | NR | M |
| ENL1 | 32 | LL | ENL | F |
| ENL2 | 35 | LL | ENL | M |
| ENL3 | 35 | LL | ENL | M |
| ENL4 | 35 | BL | ENL | M |
| ENL5 | 50 | LL | ENL | M |
| ENL6 | 48 | LL | ENL | F |
| ENL7 | 46 | LL | ENL | M |
| ENL8 | 74 | LL | ENL | M |
| ENL9 | 42 | LL | ENL | M |
| ENL10 | 63 | LL | ENL | M |
| ENL11 | 41 | LL | ENL | F |
| ENL12 | 45 | LL | ENL | M |
| ENL13 | 29 | LL | ENL | M |
| ENL14 | 39 | LL | ENL | M |

ID: randomized code for each patient in order to safeguard their identity; Form of Disease: LL = lepromatous leprosy, ENL = erythema nodosun leprosum; Gender: M = male, F = female.
