# Supplementary material for: Blood coagulation abnormalities in multibacillary leprosy patients
Source: PLoS Negl Trop Dis. 2018 Mar 22;12(3):e0006214. doi: 10.1371/journal.pntd.0006214 (PMC5863944; doi:10.1371/journal.pntd.0006214)
Supplement: S2 Table — (DOCX) [file pntd.0006214.s002.docx]

**Supplementary Table 2 - Identification of leprosy patients which presented the Leprosum Clot (LC+)**

| **ID** | **Age (years)** | **Form of disease** | **Reaction** | **Gender** |
| --- | --- | --- | --- | --- |
| **LCP1** | 28 | LL | ENL | M |
| **LCP2** | 62 | LL | ENL | M |
| **LCP3** | 63 | LL | ENL | M |
| **LCP4** | 55 | LL | ENL | M |
| **LCP5** | 37 | BL | T1R | M |
| **LCP6** | 66 | LL | ENL | M |
| **LCP7** | 39 | LL | ENL | M |
| **LCP8** | 60 | BT | T1R | F |
| **LCP9** | 36 | LL | ENL | F |
| **LCP10** | 38 | LL | ENL | M |
| **LCP11** | 38 | BL | ENL | M |
| **LCP12** | 41 | BT | T1R | M |
| **LCP13** | 45 | LL | ENL | M |
| **LCP14** | 40 | LL | N/R | M |
| **LCP15** | 56 | LL | N/R | M |
| **LCP16** | 33 | LL | N/R | M |
| **LCP17** | 71 | LL | N/R | M |
| **LCP18** | 34 | LL | ENL | F |
| **LCP19** | 66 | LL | N/R | F |
| **LCP20** | 56 | BT | N/R | F |
| **LCP21** | 76 | LL | N/R | F |
| **LCP22** | 57 | BT | N/R | F |
| **LCP23** | 50 | LL | N/R | M |
| **LCP24** | 70 | BB | T1R | F |
| **LCP25** | 53 | LL | N/R | F |
| **LCP26** | 32 | LL | N/R | F |
| **LCP27** | 50 | LL | N/R | M |
| **LCP28** | 66 | LL | N/R | F |
| **LCP29** | 10 | LL | N/R | M |
| **LCP30** | 30 | BL | N/R | M |
| **LCP31** | 40 | LL | ENL | M |
| **LCP32** | 68 | LL | N/R | F |
| **LCP33** | 48 | LL | N/R | M |
| **LCP34** | 43 | LL | N/R | M |
| **LCP35** | 30 | LL | N/R | F |

ID: randomized code for each patient in order to safeguard their identity; Form of Disease: BT = borderline tuberculoid, LL = lepromatous leprosy, BL = borderline lepromatous, BB = borderline borderline, T1R = type I reaction, ENL = erythema nodosun leprosum, N/R = non reactional; Gender: M = male, F = female.
