# Supplementary figures and images for: Blood coagulation abnormalities in multibacillary leprosy patients
Source: PLoS Negl Trop Dis. 2018 Mar 22;12(3):e0006214. doi: 10.1371/journal.pntd.0006214 (PMC5863944; doi:10.1371/journal.pntd.0006214)

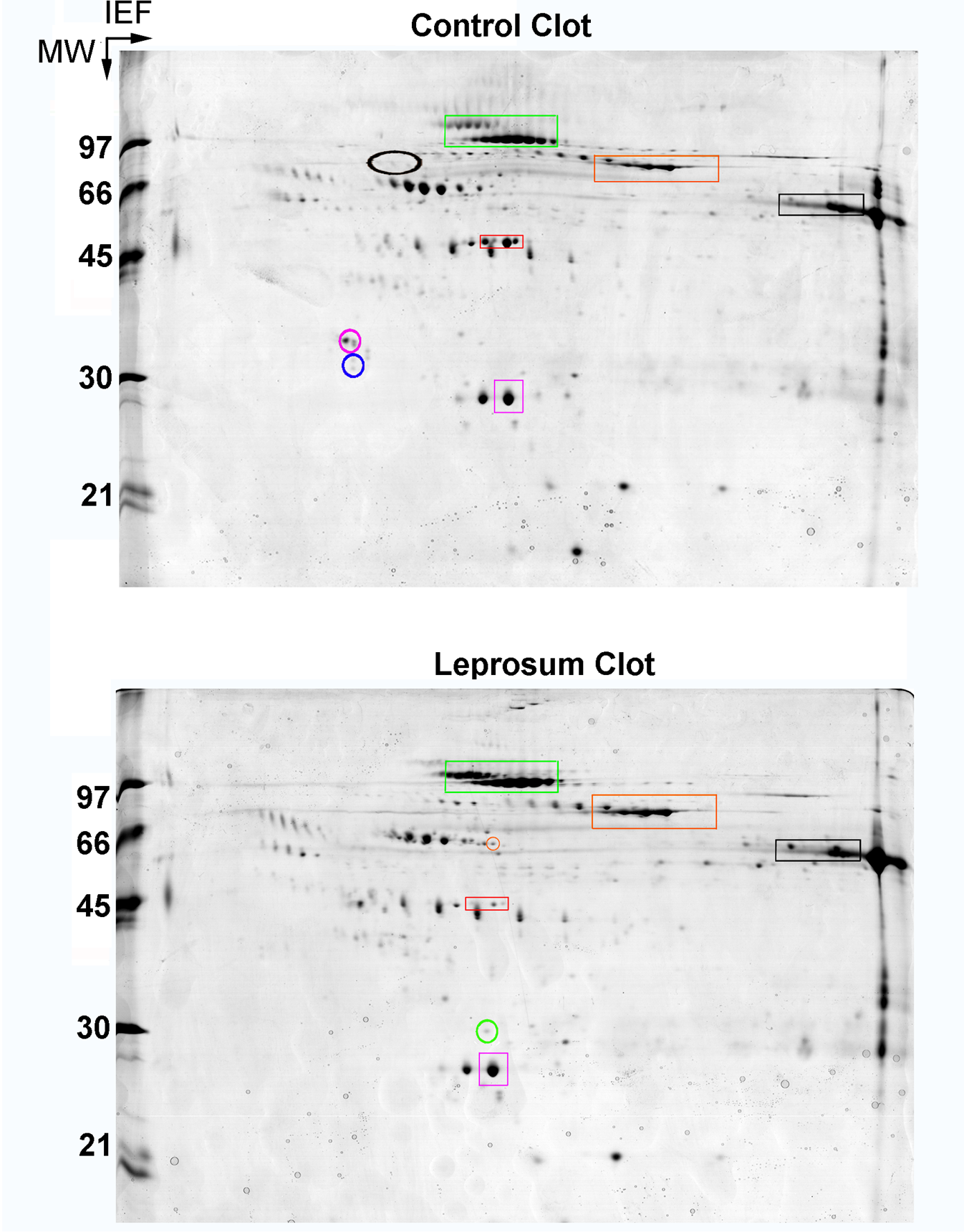

Supplement: S1 Fig — 2D gel differential analysis between a control and leprosum clot protein fractions on IPG strips covering pH 4–7. Representative gels stained with Coomassie colloidal blue were spots were trypsin-digested and identified by MALDI-TOF. Spots delimited by an orange box correspond to alpha fibrin; black box correspond to beta fibrin; green box correspond to gamma fibrin; purple box correspond to apolipoprotein A1 and red box correspond to beta actin. Proteins exclusively found in control or leprosum clot are identified with circles as follow: black circle correspond to kininogen 1, purple circle correspond to Tropomyosin alpha-4, blue circle correspond to Tyrosine 3-monooxygenase, orange circle correspond to IHRP and green circle correspond to Complement C4. Arrows indicate the direction of separation by isoelectric point (IEF) and molecular weight (MW). (TIF) [file pntd.0006214.s003.TIF]

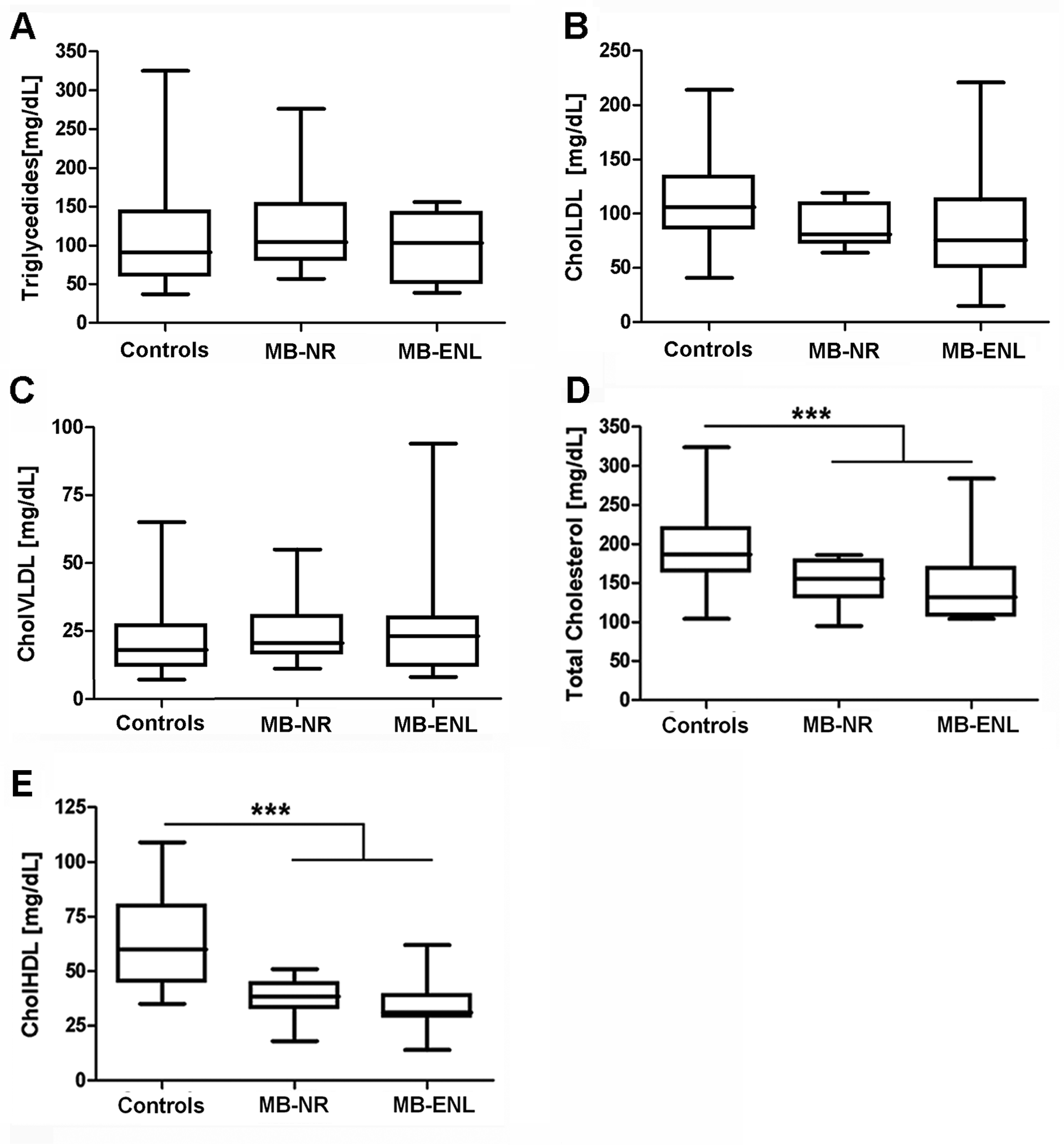

Supplement: S2 Fig — Total plasma triglycerides (A), LDL-cholesterol (B) and VLDL-cholesterol (C) levels were determined for 50 non-leprosy patients plasma (controls), MB-NR group are composed by 10 LL and 1 BL patients. MB-ENL group are composed by 13 LL and 1 BL individuals. D) Total plasma cholesterol and HDL-cholesterol (E) determined for 50 non-leprosy patients plasma (controls), MB-NRgroup are composed by 10 LL and 1 BL patients. MB-ENL group are composed by 13 LL and 1 BL individuals.*** indicates p<0.0001 with ANOVA. (TIF) [file pntd.0006214.s004.tif]
